# Exogenous salicylic acid regulates organic acids metabolism in postharvest blueberry fruit

**DOI:** 10.3389/fpls.2022.1024909

**Published:** 2022-10-17

**Authors:** Bo Jiang, Xiangjun Fang, Daqi Fu, Weijie Wu, Yanchao Han, Hangjun Chen, Ruiling Liu, Haiyan Gao

**Affiliations:** ^1^ College of Food Science and Technology, Nanjing Agricultural University, Nanjing, China; ^2^ Food Science Institute, Zhejiang Academy of Agricultural Sciences, Hangzhou, China; ^3^ Key Laboratory of Post-Harvest Handling of Fruits, Ministry of Agriculture and Rural Affairs, Hangzhou, China; ^4^ Key Laboratory of Fruits and Vegetables Postharvest and Processing Technology Research of Zhejiang Province, Hangzhou, China; ^5^ Key Laboratory of Postharvest Preservation and Processing of Fruits and Vegetables, China National Light Industry, Hangzhou, China; ^6^ College of Food Science and Nutritional Engineering, China Agricultural University, Beijing, China

**Keywords:** salicylic acid, organic acid metabolism, blueberry fruit, postharvest storage, organoleptic quality

## Abstract

Fruit acidity is an essential factor affecting blueberry organoleptic quality. The organic acid content in blueberry fruit mainly contributes to fruit acidity. This study aims to evaluate the effect of exogenous salicylic acid (SA), the principal metabolite of aspirin, on the organoleptic quality and organic acid metabolism in rabbiteye blueberry (*Vaccinium virgatum* Ait, ‘Powderblue’) during cold storage (4 °C). Results showed that SA-treated fruit reduced fruit decay and weight loss delayed fruit softening, and decline of total soluble solids (TSS). TA and total organic acid amounts stayed the same during the late storage period in SA-treated fruit. Four kinds of organic acid components, malic acid, quinic acid, citric acid, and succinic acid, were at higher levels in fruit treated by SA as compared to control. SA enhanced the activities of PEPC, NAD-MDH, and CS to promote the synthesis of malic acid and citric acid. Meanwhile, the activities of NADP-ME, ACL, and ACO, which participated in the degradation of malic acid and citric acid, were inhibited by SA. qPCR results also showed that the expression of *VcPEPC*, *VcNAD-MDH*, and *VcCS* genes were upregulated. In contrast, SA downregulated the expression of *VcNADP-ME*, *VcACL*, and *VcACO* genes. In conclusion, SA could regulate the key genes and enzymes that participated in organic acids metabolism to maintain the freshness of blueberry during cold storage, therefore minimizing the economic loss.

## Introduction

Blueberry, the *Vaccinium* genus, is popular with consumers because of its high nutritional and healthy benefits. Fruit acidity is an essential component affecting fruit quality and edible flavor (Etienne et al., 2013). Fruit acidity is mainly affected by the accumulation of organic acids (Giné Bordonaba and Terry, 2010). The type and proportion of main organic acids in fruit differ from species and cultivars (Mark et al., 1994). For instance, citric acid is the primary organic acid in ponkan (Gao et al., 2018) and tomato (Yan et al., 2021) fruit. Malic acid is dominant in apples (Zhu et al., 2022), pears (Wu et al., 2022), and peach (Yang et al., 2020) fruit. The content of organic acids varied during fruit development and storage (Das et al., 2022; Dragišić Maksimović et al., 2022). In the three maturation stages of blueberry fruit (green, red, and blue), the total acid decrease with the fruit ripening (Li et al., 2020). Five kinds of organic acid identified from O’ Neal blueberry fruit (*V. corymbosum*) all dropped from green to blue stage. The levels of various organic acids in blueberry also showed the different trend during the storage (Nguyen et al., 2021; Smrke et al., 2021).

The change of organic acids in fruit is attributed to acid balance between acid synthesis and degradation (Zheng et al., 2021). The tricarboxylic acid (TCA) cycle is the main pathway to synthesizing organic acids, such as malic acid and citric acid (Huang et al., 2021). Malic acid accumulation and degradation were regulated by the activities of NAD-dependent malate dehydrogenase (NAD-MDH, EC 1.1.1.37), cytosolic NADP-dependent malic enzyme (NADP-ME, EC1.1.1.40), and phosphoenolpyruvate carboxylase (PEPC, EC4.1.1.31) (Liao et al., 2022). NAD-MDH and PEPC activities are responsible for the malic acid biosynthesis, while NADP-ME relates to malic acid degradation (Liu et al., 2016). As for the citric acid, citrate synthase (CS, EC, 2.3.3.1), aconitate hydratase (ACO, EC 4.2.1.3), and ATP-citrate synthase (ACL, EC 4.1.3.8) participated in the citrus metabolism. One of citrate degradation is a γ-Aminobutyric acid (GABA) shunt that citric acid is catalyzed to form succinic acid (Li et al., 2020). Another pathway is to join in the transformation of malic acid by forming OAA and acetyl-CoA under the action of ACL (Guo et al., 2020).

Salicylic acid (SA), the principal metabolite of aspirin, crucial endogenous hormone, participated in fruit quality formation and disease resistance against the pathogen (Rachappanavar et al., 2022). In recent years, exogenous SA treatment has been shown to maintain postharvest fruit quality and delay fruit decay and disease incidence (Yang et al., 2020; Yang et al., 2022). Moreover, SA treatment promoted the accumulation of organic acid in citrus fruit (Wang et al., 2020) and jujube fruit (Yang et al., 2022). However, another study indicated that organic acids were no significant difference in peach fruit between SA treated and control fruit (Yang et al., 2020). Therefore, the effect of exogenous SA on organic acid metabolism needs to be further studied. In addition, our previous study found that SA affected the titratable acidity (TA) level in blueberry fruit during ambient temperature storage (25 °C) (Jiang et al., 2022). However, the change regularity and metabolism mechanism of organic acid under SA treatment in postharvest blueberry fruit was still less reported. This paper studied the change of organic acids and their metabolism related enzymes and genes expression levels in postharvest blueberry treated by exogenous SA. These results may provide some evidence for revealing the regulation of SA on organic acid mechanism in blueberry stored in cold condition (4°C).

## Materials and methods

### Experimental materials

The mature rabbiteye blueberries (*Vaccinium virgatum* Ait, ‘Powderblue’) were harvested from a commercial orchard located in Xinchang, Zhejiang Province, on July 15, 2021. The same maturity, uniform size, no disease, and no mechanical damage, blueberry was selected to slip back to the laboratory for subsequent experiments.

### Treatment and sampling

All berries were divided randomly into two groups (SA group and control group). SA group was soaked in 1.0 mmol L^-1^ SA solution for 5 min (SA group). Control group was soaked with sterile water for 5 min. Each group was performed in triplicate. The treated berries were air-dried and packed in a plastic box with polyethylene terephthalate. Each box of 125 g berries was stored at 4 °C for 30 days.

Fruit quality was analyzed at six days intervals during cold storage. Forty-five berries were taken from each replication at random for measurement of fruit decay and weight loss every six days, respectively. ten berries were selected for the determination of fruit firmness. Ten berries were sampled for total soluble solids (TSS) and TA. The remaining berries per replicate were cut into small pieces with a scalpel and frozen quickly in liquid nitrogen. The sample pieces were stored in -80 °C for assays of organic acids metabolism.

### Analysis of fruit quality

The decay incidence was evaluated according to the number of berries with rot, lesion, or visible pathogen growth among all berries. The result was presented as the percentage (%) of decay berries with respect to the total number of berries. The weight loss was calculated by weighing berries before and after storage. The result was evaluated as the percentage (%) of weight reduction relative to the original weight. The fruit firmness was measured by a texture analyzer (TA-XT Plus, Stable Micro Systems Ltd, Surrey, U.K.) equipped with a probe of 6 mm diameter. The experimental parameters were as follows. Compression position: fruit equator; compression distance: 5 mm; measuring speed: 1.0 mm s^-1^. The firmness was presented as the maximum pressing force (N). TSS content in berries was recorded by a digital refractometer (PAL-1, Atago, Tokyo, Japan), and the result was expressed as a percentage (%). TA in berries was measured by an automatic titrator (877 Titrino plus, Metrohm, Herisau, Switzerland), and the result was expressed as a percentage of malic acid (%).

### Determination of organic acids

Determination of organic acids was conducted following the method of Li et al. (2020). Approximately 2 g frozen sample (ground powder) was added into 2mL ultrapure water and then extracted by ultrasonic irradiation for 30 min. The mixture was centrifuged at 8000 ×g for 10 min (4 °C). The supernatant was collected into a centrifuge tube of 10 mL, and the residue was extracted again with ultrapure water of 1 mL. The second extract was centrifuged at 8000 ×g for 10 min (4 °C). The supernatant centrifuged twice were mixed and filtered through a 0.22 μm microporous membrane filter. The filtrate was used for the analysis of organic acids.

The composition and content of organic acids was analyzed by HPLC. The HPLC analysis was performed by Waters Alliance 2995 system (Waters Corporation, Milford, USA) with an RP-HPLC XDB-C18 column (5 μm,250 mm×4.6 mm, Agilent, CA, USA). The mobile phase was 0.01 M KH_2_PO_4_ buffer (pH=2.7) with 4% (v: v) methanol. The flow rate was 0.6 mL/min. The temperature of the column was set at 25°C. The wavelength of the ultraviolet detector (2998, Waters Corporation, Milford, USA) was set at 210 nm. The injection volume was 10 μL. Organic acid was quantified by different external standards purchased from Sigma-Aldrich (St. Louis, MO, USA). The retention time, calibration curves and correlation coefficients of external standards were showed in **Supplementary Table S1**.

### Extraction and assay of enzyme activities.

Crude enzymes of PEPC, NAD-MDH, NADP-ME, and CS were extracted according to the method of Millaleo et al. (2019). PEPC, NAD-MDH, NADP-ME, and CS activity were measured according to Chen et al. (2009). ACL and ACO were extracted and assayed following the method of Guo et al. (2020). All enzyme activities were described as Unit (U) kg^−1^ fresh weight (FW).

### Extraction of RNA and synthesis of cDNA

Total RNA was extracted from 150 mg frozen samples (ground powder in liquid nitrogen) using an RNAprep Pure Plant Kit (Polysaccharides & Polyphenolics-rich) (Tiangen, Beijing, China). The concentration and purity of total RNA were assayed using a BioSpec-nano spectrophotometer (Shimadzu Corporation, Tokyo, Japan). The first-strand cDNA was synthesized using a StarScript II First-strand cDNA synthesis kit with gDNA Remover (GeneStar, Beijing, China)

### Quantitative real-time PCR analysis

The qRT-PCR primers were designed using Primer Premier 5.0 software (Premier Biosoft International, San Francisco, USA) and synthesized by Youkang Biotech (Hangzhou, China). The specific primers sequences of organic acid metabolism-related genes were shown in **Table 1**. The qRT-PCR analysis was operated by using 2×RealStar Green Fast Mixture with ROX (GeneStar, Beijing, China) and a quantitative fluorescence analyzer (StepOne Plus, ABI, Waltham, USA). The qPCR procedure was as follows: predenaturation at 95°C for 2 min, followed by a cycle reaction (40 cycles) of 95 °C for 15s, 55 °C for 30 s. Melting curve was preformed from 55 to 95 °C at an increasing speed of 0.5°C per second. The relative gene expression levels were evaluated using the ABI’s Stepone 2.3 software and the 2^-ΔΔCt^ method (Livak and Schmittgen, 2001). The GAPDH gene (gene accession number: AY123769) was served as the internal reference gene to normalize the expression data. All qRT-PCR reactions were performed in triplicate.

**Table 1:** The sequences of primers used for qPCR.

Gene	GeneID	Forward primer sequence (5’-3’)	Reverse primer sequence (5’-3’)
*VcPEPC*	KT995478	CCGCCTTCGTGACTCCTACAT	TGCTATCGCCTATGAAGTCC
*VcNAD-MDH*	Vadar_g11215	ACGATCTGTTCAACATCAATGC	GCTCCCAGTACTTTGATTTTGG
*VcNADP-ME*	Vadar_g15676	CTGATGGCGAGCGGATTTTG	TATGGGCAGGCACGAAGAAG
*VcCS*	DR067095	GTAGACACGGTGCCCAAATC	TCATGGTGGAGCAAATGAAG
*VcACL*	MH048701	GGCTCAACTCTTTCGGACCA	CAGGGCTTCCACAAGGGAAT
*VcACO*	Vadar_g34681	TGGCAAGAGGAACTTTCGCA	CTGCTCCGGCCAAGACAATA
*VcGAPDH*	AY123769	GCTGTACCACAAACTGTCTTGC	ATGAAGCAGCTCTTCCACCTCT

### Statistical analysis

Statistical analysis was conducted using SPSS 19.0 statistical software package (IBM Corporation, Chicago, USA). The significant difference was carried out using One-way analysis of variance (ANOVA) combined with Duncan’s multiple-range test (P < 0.05), Figures were plotted with Origin 9.4 (OriginLab, Northampton, USA). Online Hiplot software (Shanghai Tengyun Biotech, Shanghai, China) (https://hiplot-academic.com/basic/corrplot) was applied to illustrate the correlation. All data are presented as mean ± standard deviation.

## Results

### Effect of SA treatment on fruit quality

**Figure 1:**
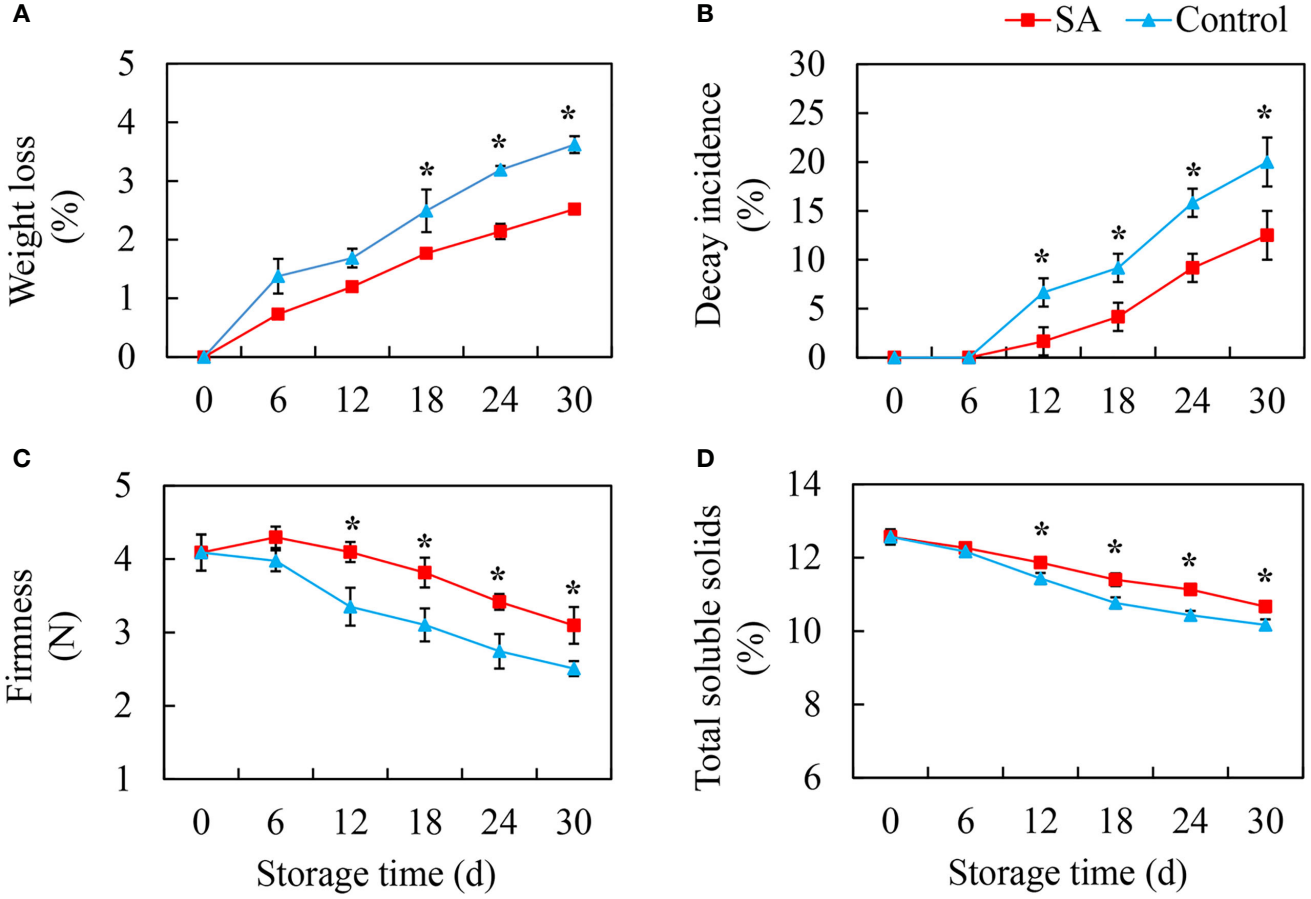
Effects of Salicylic acid treatment on fruit quality of blueberry during cold storage. **(A)**, Weight loss; **(B)**, Decay incidence; **(C)**, Firmness; **(D)**, Total soluble solids (TSS). Error bars represent the standard deviations of the means (n=3). The asterisks show a significant difference between SA-treated and control fruit (*P* < 0.05).

The weight loss of fruit showed an increasing trend during cold storage. Compared to the control fruit, SA-treated fruit had lower weight loss (**Figure 1A**). The decayed fruit began to appear after 12 days of storage, and the decay incidence in the control fruit reached 20.0% at 30 d, while decay incidence in SA-treated fruit was only 12.5% (**Figure 1B**). The change in firmness was opposite to the change in weight loss. The fruit becomes softer after storage. SA treatment could delay the decrease of fruit firmness (**Figure 1C**). The content of TSS exhibited a downward trend, and the differences between SA-treated and control fruit appeared after 12 days and 18 days of storage, respectively (**Figure 1D**).

### Effect of SA treatment on TA and organic acids

**Figure 2:**
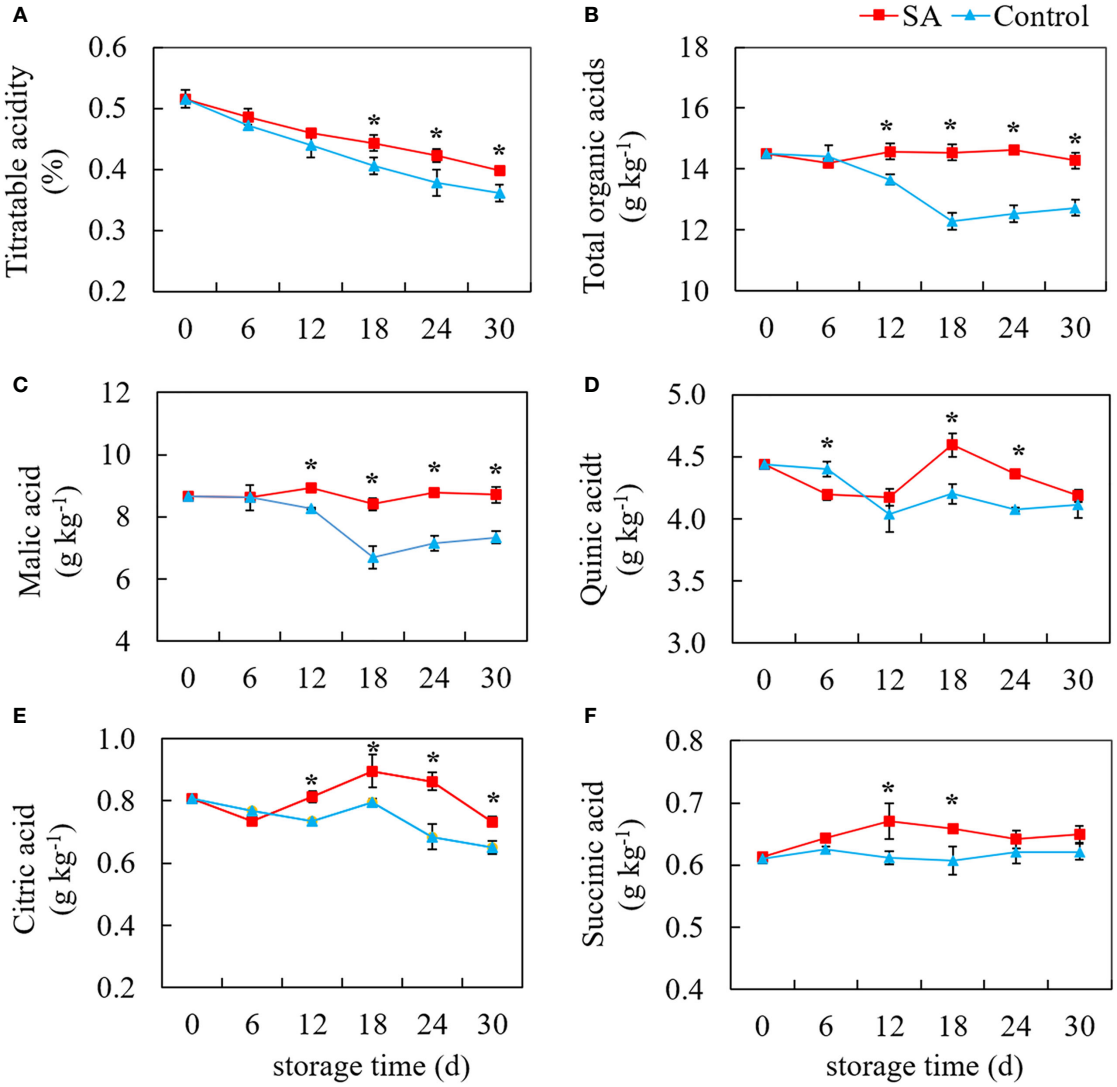
Effects of Salicylic acid treatment on organic acid of blueberry during cold storage. **(A)**, Titratable acidity (TA); **(B)**, total organic acid; **(C)**, Malic acid; **(D)**, Quinic acid; **(E)**, Citric acid; **(F)**, Succinic acid. Error bars represent the standard deviations of the means (n=3). The asterisks show a significant difference between SA-treated fruit and control fruit (*P* < 0.05).

As shown in **Figure 2**, TA level in fruit displayed a declining trend during cold storage. Compared with control, the TA in SA-treated fruit was higher during the late stage (After 18 days) (**Figure 2A**). The change of total organic acids, not completely consistent with the change of TA, showed a tendency to decrease first and then remain unchanged (**Figure 2B**). In comparison, SA treatment maintained the total organic acid to some degree. Malic acid was the highest organic acid content in ‘Powderblue’ blueberry, followed by quinic acid, citric acid, and succinic acid. Malic acid content in control fruit decreased from 8.66 g kg^-1^ at 0 d to 6.70 g kg^-1^ at 18 d, and then slightly decreased to 7.34 g kg^-1^ at 30 d. While malic acid in SA-treated fruit showed no obvious change during storage (**Figure 2C**). Compared with the control fruit, quinic acid in SA-treated fruit great declined after 6 days, but after 12 days, quinic acid content increased first and then decreased (**Figure 2D**). Although the content of citric acid in the control fruit decreased during storage, there was a high peak value at 18 d (**Figure 2E**). SA treatment increased the content of citric acid during the mid and late periods. Succinic acid was the lowest of the four organic acids, accounting for 4.22% of total organic acid. There was no significant change in the succinic acid content of control during storage, but succinic acid in SA-treated fruit was an obvious increasing trend before 18 days (**Figure 2F**). These results showed SA treatment might mediate blueberry’s organic acid metabolism after harvest.

### Effect of SA treatment on enzymes activities participating in organic acids metabolism

**Figure 3:**
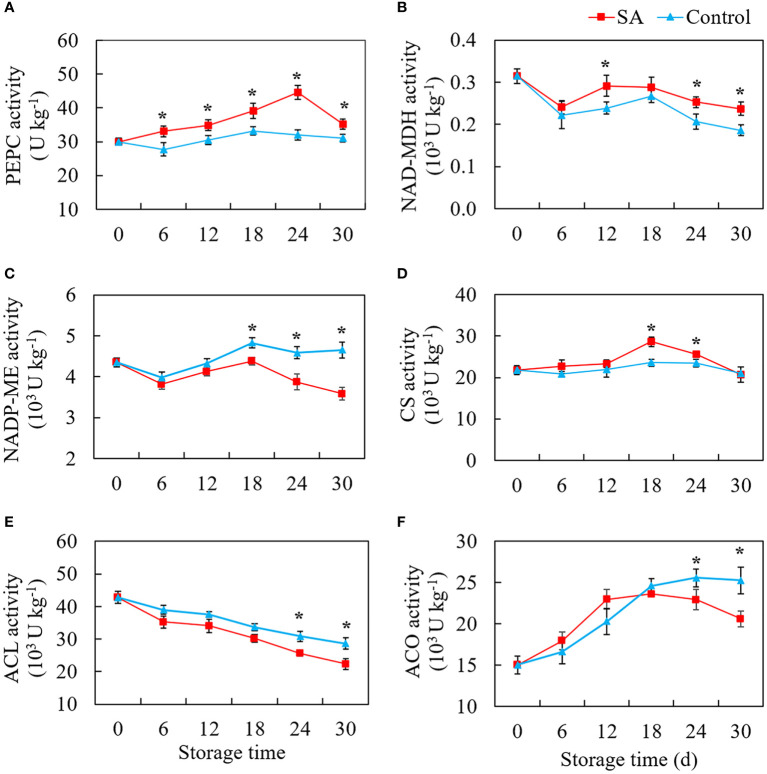
Effects of salicylic acid treatment on enzyme activities participated in organic acid metabolism of blueberry during cold storage. **(A)**, phosphoenolpyruvate carboxylase (PEPC); **(B)**, NAD-dependent malate dehydrogenase (NAD-MDH); **(C)**, NADP-dependent malic enzyme (NADP-ME); **(D)**, Citrate synthase (CS); **(E)**, ATP-citrate synthase (ACL); **(F)**, cytoplasm aconitase (ACO). Error bars represent the standard deviations of the means (n=3). The asterisks show a significant difference between SA-treated fruit and control fruit (*P* < 0.05).

**Figure 3** shows the change patterns of three malic acid metabolism-related enzymes (PEPC, NAD-MDH, and NADP-ME) and three citric acid metabolism-related enzymes (CS, ACO, and ACL) in SA treated and control fruit during cold storage. PEPC activity in the control fruit was no significant change with the extension of storage time (**Figure 3A**). But SA-treated fruit maintained higher PEPC activity as compared to control. The activity of NAD-MDH declined from 0.31 U kg^-1^ at 0 d to 0.24 U kg^-1^ and 0.22 U kg^-1^ at 6 d in SA-treated and control fruit, respectively (**Figure 3B**). But an increase in NAD-MDH activity in control fruit was observed at the -mid-stage (from 12 to 18 days) and then decreased to 0.19 U kg^-1^ at 30 d. Although the change of enzyme activity was similar to that in control, NAD-MDH activity in SA-treated showed higher levels during the late period (24 days and 30 days). Considering the level of NADP-ME activity, a drop of activity was observed in the first six days, and activity climbed to the highest peak at 18 d (**Figure 3C**). While during the late storage, the fruit showed a significant decline in NADP-ME activity, and the fruit treated by SA had a higher NADP-ME activity than the control fruit. As for three enzymes that participated in citric acid accumulation, control fruit had no obvious fluctuation in CS activity during whole storage, while the CS activity in SA-treated fruit showed an increasing trend after 12 days and then declined to the same level in control at 30 d (**Figure 3D**). The ACL activity decreased overall during storage both in SA-treated and control fruit. The higher enzyme activities happened in fruit with SA treatment at 6 d and 30 d as compared with control (**Figure 3E**). The activity level of ACO in fruit reached a higher level from 15.01 U kg^-1^ at 0 d to 24.58 U kg^-1^ at 18 d during the early storage and then remained stable for 30 days (**Figure 3F**). SA treatment reduced ACO activity during the late storage.

### Effect of SA treatment on genes expression involved in organic acids metabolism

**Figure 4:**
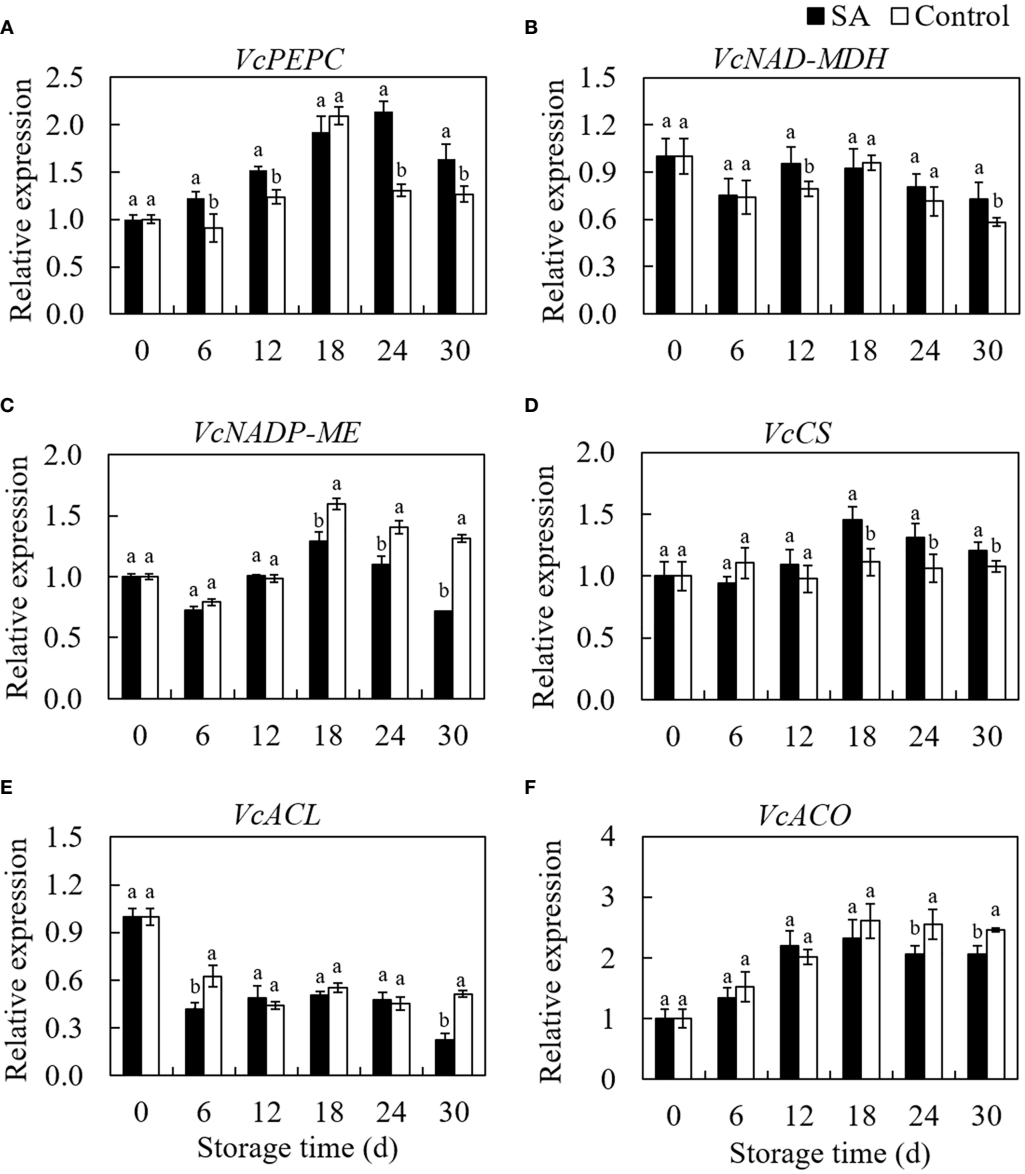
Effects of Salicylic acid treatment on genes expressions involved in organic acid metabolism of blueberry during cold storage. **(A)**, *VcPEPC*; **(B)**, *VcNAD-MDH*; **(C)**, *VcNADP-ME*; **(D)**, *VcCS*; **(E)** *VcACL*; **(F)**, *VcACO*. Error bars represent the standard deviations of the means (n=3). The different lowercase letters indicate the significant differences between SA-treated and control fruit at each sampling time (P<0.05).

*VcPEPC* gene expression increased from 0 d to 18 d then declined to 30 d in control fruit during storage (**Figure 4A**). Even if similar to the changing trend of control, the SA-treated fruit showed a more extended increasing period from 0 d to 24 d. SA treatment promoted the *VcPEPC* expression during whole storage, except at 18 d. Fluctuation of the gene expression level of *VcNAD-MDH* in SA-treated and control fruit was consistent with storage time (**Figure 4B**). SA-treated fruit was higher in *VcNAD-MDH* expression at 12 d and 30 d compared to control. regarding the transcript level of *VcNADP-ME* in blueberry, a higher expression in SA-treated fruit was observed than that in control during the end of storage (from day 18 to day 30) (**Figure 4C**). The transcription of *VcCS* was no significantly changed in control during storage, while SA treatment upregulated the expression of *VcCS* in blueberry after 12 days of storage (**Figure 4D**). *VcACL* expression in control showed a decreasing trend at the early stage (before 12 days) and remain stableto 24 d (**Figure 4E**). It is noteworthy that *VcACL* expression considerably rose from 24 d to 30 d (about 1.80 times). Fruit treated by SA still was in a lower level of *VcACL* at 30 d. The *VcACO* expression increased by 2.61 times from 0 d to 18 d in control and then declined slightly to 30 days (**Figure 4F**). The change trend in SA-treated fruit was similar to the change in control, but lower expression levels were observed than that in control during the late storage. These results indicated that SA treatment could regulate the expression of key genes involved in the organic acid metabolism of blueberry.

### Correlation analysis of indicators involved in organic acid metabolism

**Figure 5:**
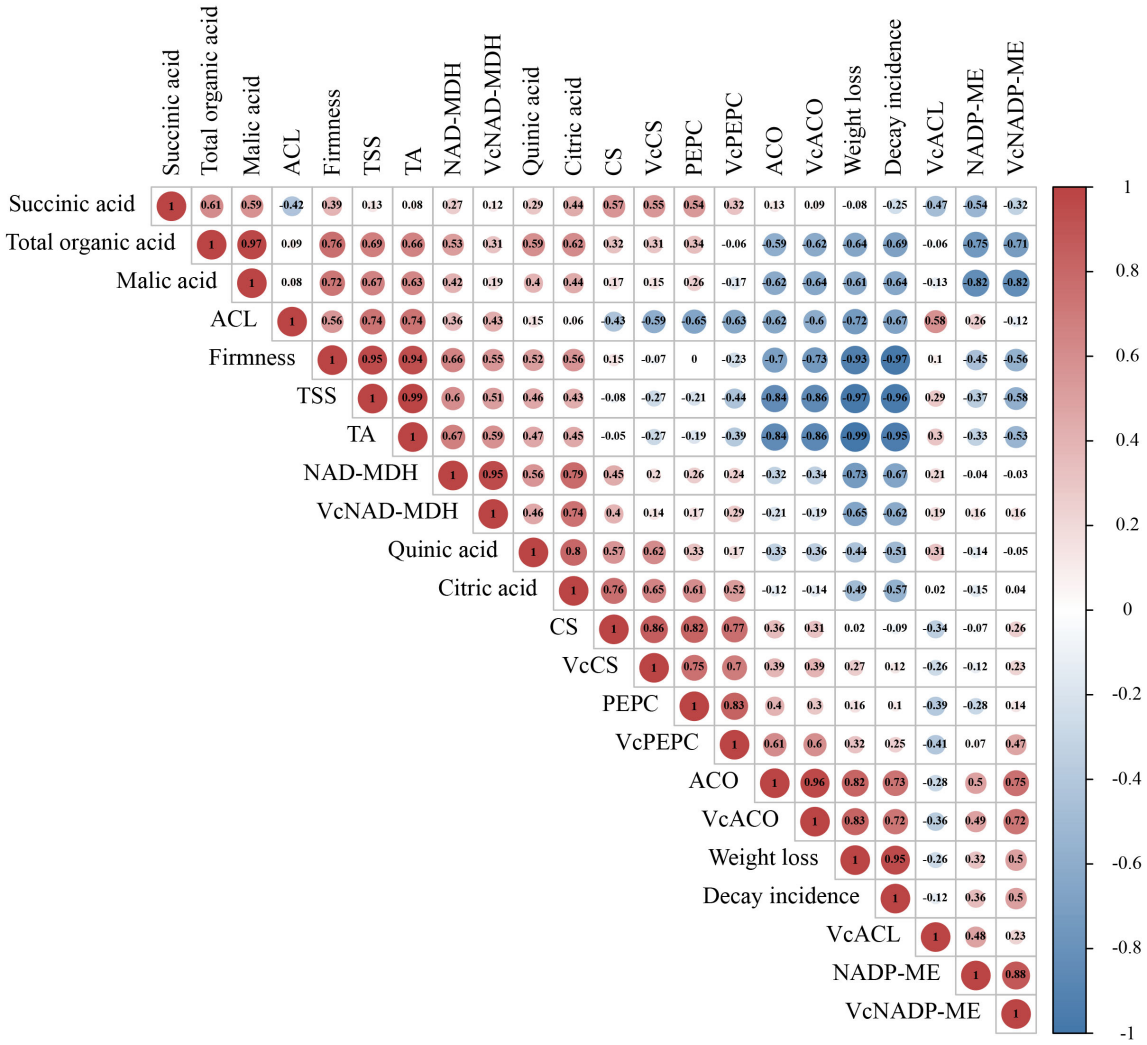
Correlation analysis of indicators involved in organic acid metabolism. The Correlation in all indicators was conducted by Pearson Correlation Analysis. The value in the circle represents the Pearson correlation coefficient between the heading of the column and the row. The closer the value is near to 1 or -1, the greater the correlation. Red color represents positive correlation; blue represents negative correlation.

Correlation analysis in all indicators involved in organic acid metabolism was conducted by the Pearson correlation coefficient. From the results in **Figure 5**, we found that the coefficient between TA and total organic acid was 0.66, suggesting that fruit acidity was closely positively related to blueberry’s organic acid content during storage. Three kinds of enzymes, NAD-MDH, PEPC, and NADP-ME, participated in the accumulation and degradation of malic acid was highly related to *VcNAD-MDH*, *VcPEPC*, and *VcNADP-ME* genes, respectively. These results implicated that these genes may regulate relevant enzyme activities to affect malic acid metabolism. The correlation between three enzymes (CS, ACO, and ACL) and three genes (*VcCS*, *Vc*ACO, and *VcACL*) involved in citric acid biosynthesis and transfer showed a similar feature. In addition, the correlation difference between total organic acids and four organic acids indicated that the contribution of four acids to total acids was not equal. Interestingly, although citric acid and malic acid could transform each other by various pathways, the correlation between them was not high (0.44). This result may indicate that malic acid content did not depend entirely on the level of citric acid. In addition, organic acid, the decay incidence and weight loss of fruit were negatively correlated with total organic acids and malic acid. The firm firmness showed positively correlation with malic acids (0.72) and citric acid (0.56).

## Discussion

The weight loss, softening, and decay of ‘Powderblue’ blueberry occurred during cold storage, and these changes also existed in the postharvest period of other blueberry cultivars (Abeli et al., 2021; Xu et al., 2021). We found that SA treatment delayed the decline of weight loss and firmness, and slowed fruit decay incidence. These results were similar to other fruits such as strawberries (Zhang et al., 2022), peach (Yang et al., 2020), and litchi (Kumari et al., 2015) treated by SA. The possible reason for maintenance of fruit quality was that exogenous salicylic acid treatment induced the increase of endogenous salicylic acid content, thereby reducing the respiratory metabolism and delaying fruit senescence, which was confirmed in jujube fruit (Yang et al., 2022).

Acidity and sweetness play essential roles in the flavor quality of fruit (Yang et al., 2020). Our results showed SA treatment effectively inhibited the decline of TSS and TA, therefore maintained the taste and quality of blueberry during storage. More importantly, organic acid, the main responsible for acidity, is also closely related to the aging process and storage performance (Etienne et al., 2013). Some citrus cultivars with high-content organic acids or organic acid degradation slowly have better storage performance. The consumption of organic acid during storage after harvest was the main reason for fruit flavor and quality decline, which brings severe economic loss to fruit production (Wang et al., 2014). The total organic acids in ‘Powderblue’ fruit gradually decreased during storage. SA treatment significantly delayed the fall down of total organic acids. The higher level of organic acid in fruit provided abundant substrates for maintaining the postharvest quality of fruit. Some studies showed that the changes of organic acids affect fruit senescence and storage characteristics (Sheng et al., 2017; Habibi et al., 2020). Moreover, malic acid and citric acid, also as the intermediate products of plant cell respiration metabolism, play an important role in plant material metabolism and energy metabolism (Etienne et al., 2013). The reduced fruit decay in the treated blueberry fruit could be ascribed to SA inhibited the degradation of organic acids, which helped to suppress respiration and resulted in lower energy consumption Meanwhile, the reduction of organic acids consumption maintained an acidic environment, which slowed down fruit senescence (Angioni and Schirra, 2011). Among four kinds of organic acids, malic acid was the predominant acid, followed by quinic and citric acid. The types and distribution of organic acids in blueberry were different in cultivars. Citric acid was the primary acid in lowbush (*Vaccinium angustifolium*) (Kalt and McDonald, 1996) and highbush blueberry (*V. corymbosum*) (Zhang et al., 2020). But the interesting thing is that quinic acid was the highest in some clones of lowbush blueberry (Gibson et al., 2013). The distribution discrepancy of organic acids may be due to diversity in blueberry genotypes and growth environment.

Although the total amount of organic acids decreased in blueberry during storage, the changes were not consistent between the four kinds of acids. These change differences in organic acids were also reported in other highbush blueberry cultivars during storage (Smrke et al., 2021; Dragišić Maksimović et al., 2022). Dragišić Maksimović et al. (2022) found that the total acids, citric acid, malic acid, and shikimic acid in ‘Bluecrop’ and ‘Liberty’ blueberry showed the decline in varying degrees with the elongation of the storage period, while quinic acid content was no significant change. In our study, SA treatment slowed the descent of malic acid in the mid and late storage period. Malic acid is the key intermediate in the TCA cycle, and synthesis and degradation of malic acid are closely related to the TCA cycle pathway (Fernie and Martinoia, 2009). Malic acid accumulation was reported to be negatively related to NADP-ME and positively related to NAD-MDH and PEPC activity in fruit (Liu et al., 2016). SA treatment was found to inhibit the NADP-ME activity and maintain the higher activity of NAD-MDH, suggesting that SA kept the balance of malic acid biosynthesis and degradation *via* regulating these enzyme activities. NAD-MDH catalyzes the reversible reactions from OAA to malic acid (Li et al., 2020). In addition, malic acid and OAA also participated in gluconeogenesis metabolism during the fruit ripening process (Etienne et al., 2013). In the gluconeogenesis pathway, OAA was formed from phosphoenolpyruvic acid under the catalysis of PEPC (Perotti et al., 2010). The higher level of OAA accumulation also increases the possibility of malate synthesis. PEPC was also positively correlated with malic acid accumulation in apples (Yao et al., 2009). The higher activity of PEPC in SA-treated blueberry suggested that the result was consistent with higher malic acid content than that in the control fruit. CS, ACL, and ACO are involved in citric acid biosynthesis and degradation. SA treatment enhanced the CS activity and inhibited ACO and ACL activity in the late period, which explained the increase of citric acid content. But the changing trend of ACL and ACO activities levels, two citrate degrading enzymes, presented the inverse trend during whole storage. These indicated that citric acid accumulation and degradation were co-regulated by several enzymes. The coregulation phenomenon often occurs in the acid metabolism of other fruits, such as apples (Liu et al., 2016) and peach (Liao et al., 2022).

The catalytic function of enzymes in organic acid metabolism requires transcription regulation by related genes. The results of qRT-PCR suggest that SA treatment upregulated the expression of the *VcPEPC* gene and downregulated *VcNADP-ME* during storage, consistent with changes in PEPC and NADP-ME activity. Similarly, a higher level in *VcNADP-MDH* gene expression and NADP-MDH activity in SA-treated fruit also demonstrated that SA maintained malic acid in blueberry after harvest by regulating the malic acid biosynthesis/degradation enzymes and related genes expression. Yang et al. (2022) also found that malic acid content in stored Jujube fruit was higher after being treated by exogenous SA. In addition, the study on citrus fruit also indicated that SA-induced differential expression of proteins participated in the TCA cycle and acid metabolism (Wang et al., 2020). Regarding citric acid metabolism, SA partially inhibited the transcript expression of *VcACL* and *VcACO genes*, leading to the lower activities of citric acid degrading enzyme activities as compared to control. Meanwhile, the upregulation of *VcCS* expression in SA-treated fruit also promoted the *CS* activity to accumulate citric acid. In addition to promoting citric acid synthesis, SA treatment affected succinic acid content in mid-storage time. citric acid degradation and succinic acid synthesis were also regulated by the GABA shunt (Fait et al., 2008). We speculated that the increase of citric acid and succinic acid in SA-treated fruit might be regulated by GABA pathway. However, how SA would affect citric acid and succinic acid in postharvest blueberry through regulating key enzymes and genes involved in the GABA pathway remains to be further studied.

## Conclusion

In our study, postharvest SA treatment reduced weight loss and decay incidence of blueberry fruit and delayed the decline of firmness and TSS, which maintained fruit sensory quality during cold storage. Exogenous SA was also effective in maintaining fruit acidity and organic acids of harvested blueberry. The decrease of four kinds of organic acid (malic acid, quinic acid, citric acid, and succinic acid) in ‘Powderblue’ fruit was inhibited by SA treatment to a different degree. SA upregulated the malic acid and citric acid biosynthesis-related enzymes (PEPC, NAD-MDH, and CS) and genes (*VcPEPC*, *VcNAD-MDH*, and *VcCS*) expression. Meanwhile, the activities of three enzymes (NADP-ME, ACL, and ACO) and expression levels of genes (*VcNADP-ME*, *VcACL*, and *VcACO*) participated in malic acid and citric acid degradation was downregulated by SA during storage. Under the co-regulation of enzymes and genes, the decline of organic acid content in SA-treated blueberry after harvest was delayed.

## Data availability statement

The original contributions presented in the study are included in the article/**Supplementary Material**. Further inquiries can be directed to the corresponding authors.

## Author contributions

BJ: Investigation, Formal analysis, Writing - Original Draft. XF: Investigation, Formal analysis, Writing - Review and Editing. DF: Methodology, Writing - Review and Editing. WW: Methodology, Writing - Review and Editing. YH: Writing - Review and Editing. HC: Writing - Review and Editing. RL: Supervision, Methodology, Writing - Review and Editing. HG: Conceptualization, Supervision, Project administration, Writing - Review.and Editing. All authors contributed to the article and approved the submitted version.

## Funding

This work was supported by the National Natural Science Foundation of China (Grant No. 31772042) and the Key Research & Development Program of Zhejiang Province (2021C02015).

## Conflict of interest

The authors declare that the research was conducted in the absence of any commercial or financial relationships that could be construed as a potential conflict of interest.

## Publisher’s note

All claims expressed in this article are solely those of the authors and do not necessarily represent those of their affiliated organizations, or those of the publisher, the editors and the reviewers. Any product that may be evaluated in this article, or claim that may be made by its manufacturer, is not guaranteed or endorsed by the publisher.
