# Supplementary material for: Exogenous salicylic acid regulates organic acids metabolism in postharvest blueberry fruit
Source: Front Plant Sci. 2022 Oct 17;13:1024909. doi: 10.3389/fpls.2022.1024909 (PMC9665327; doi:10.3389/fpls.2022.1024909)
Supplement: Supplementary file 1 [file Table_1.docx]

Supplementary Material

**Table S1.** The retention time, calibration curves and correlation coefficients of external standards for HPLC analysis.

| Standards | Retention time  (min) | Calibration curves | Correlation coefficients (r^2^) | Linear range  (mg mL^-1^) |
| --- | --- | --- | --- | --- |
| Quinic acid | 4.284 | y = 792,826 x + 14,244 | 0.9995 | 0.01~1 |
| Malic aicd | 5.055 | y=675,582.5 x - 7207 | 0.9999 | 0.01~1.5 |
| Citric acid | 7.75 | y = 763,964 x – 16,587 | 0.9999 | 0.01~1 |
| Succinic acid | 10.139 | y = 1,831,293 x - 71,988 | 0.9998 | 0.01~1.5 |

Note: y, peak area; x, concentration of external standard (mg mL^-1^).
